# Reply to Theeuwes: Fast Feature-based Top-down Effects, but Saliency May be Slow

**DOI:** 10.5334/joc.23

**Published:** 2018-05-14

**Authors:** Stefanie I. Becker

**Affiliations:** 1 School of Psychology, The University of Queensland, Brisbane, AU

**Keywords:** Attention, Eye movements, Visual search

In this review paper, Theeuwes (2018) continues to defend his view that (1) many effects previously believed to reflect top-down processes are in fact bottom-up/stimulus-driven, and (2) that top-down processes play a role only at a later stage of processing. This view contrasts with current models of attention, which commonly assume that both bottom-up, saliency-based processes and top-down, feature-based processes influence early processes. In these models, top-down selection is assumed to occur via the modulation of sensory neurons (organized in *feature maps*) that respond to specific elementary features (e.g., red, or tilted; e.g., Wolfe, 1994; Navalpakkam & Itti, 2007). Specifically, the intention to find a specific target such as a red item selectively increases the signals of all red items so that they have the highest activation in the attention guiding priority map (e.g., Wolfe, 1994). Importantly, this gain increase occurs in response to an *expectation* or *intention*, and can precede the appearance of the target (e.g., Kastner et al., 1999). With this, top-down processes modulate neurons at a very early point in time, even before a stimulus appears.

With regard to these models, it seems that Theeuwes does not deny that feature-specific neurons can modulate selection, but he implies that it is impossible to modulate these neurons ‘at will’ (prior to selecting the item). Instead, he maintains that (1) most feature-based effects are effects of the selection history (e.g., priming effects), and (2) that these priming effects occur automatically and are impervious to top-down control.

However, in an eye movement study, Becker, Ansorge and Horstmann (2009) showed that priming can only account for a small portion of top-down selectivity for the target (i.e., 28ms of a 76ms effect). Moreover, priming effects usually display ‘contingent automaticity’ in that they are (1) stronger or only present for task-relevant features of the target (e.g., Becker, 2010) and (2) are smaller or eliminated when participants know that the next target will have a different feature (e.g., Folk & Remington, 2008; Fecteau, 2007). So it is neither the case that priming can fully explain top-down feature-based effects, nor that priming is impervious to top-down control. Admittedly there are experiments that show no or only negligible effects of top-down knowledge (e.g., Belopolsky et al., 2010). Still, we have to keep in mind that participants do not always follow optimal strategies (e.g., Irons & Leber, 2016), and that experiments can only inform us about what people do and don’t do – not necessarily about what they can and can’t do.

**Figure 1:**
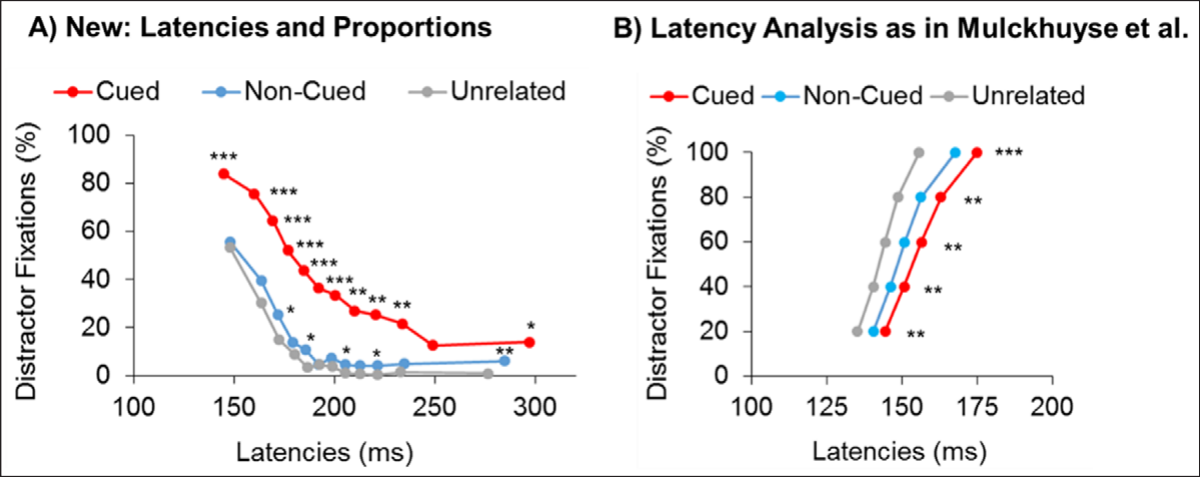
**(A)** The proportion and latencies of first eye movements to distractors that matched a word-cue announcing the target colour (Cued), mismatched the word cue but had a possible target colour (Non-cued) and distractors with a non-target colour (Unrelated). There were no differences in the timing of the 8% fastest saccades (12 bins), but a higher proportion of eye movements to the distractor that had the upcoming target colour. **(B)** Analysing the data in the same way as in Mulckhuyse et al., showed the typical results pattern of longer latencies for the earliest 20% of saccades to the cued distractor than to the non-matching or unrelated distractor (due to the fact that the distribution of the cued distractor has a longer tail; see Becker et al., 2017, Exp. 2).

A second set of studies that Theeuwes frequently cites to argue for ‘fast bottom-up and late top-down selection’ are studies where eye movement latencies are used to track the dynamic activation pattern on the priority map. For example, in a visual search task for a coloured (e.g., red) target, Mulckhuyse, van Zoest and Theewues (2008) measured the onset latency of eye movements to different types of irrelevant distractors. The typical finding is that the 20% fastest eye movements to a salient, target-dissimilar distractor (e.g., green) occur earlier in time (with a latency of ~130 ms) than the 20% fastest eye movements to the target-similar distractor (e.g., red; ~140 ms; see Figure 1B). From this it is then concluded that saliency signals dominate selection at an early stage of visual search and that top-down control enters search only at a later stage.

Becker, Lewis and Axtens (2017) however criticised that this research question requires an analysis that considers both the *timing* of eye movements and the *proportion* of eye movements to each distractor (as there are typically far fewer eye movements to the target-dissimilar distractor than the similar distractor). When both of these factors are considered, the results show that *all* eye movements, including the earliest ones, are more likely to be directed to the target-similar distractor than a target-dissimilar distractor, without any differences in the timing of early eye movements (see Figure 1A for an illustration). In a second experiment, Becker et al., randomly varied the target colour (e.g., between red and green) and used a word cue to announce the target colour on the next trial (e.g., “RED”). The results showed significantly higher selection rates of distractors matching the word cue than other distractors, including in the earliest eye movements (see Figure 1A, B). These results could not be explained by priming effects, demonstrating that top-down knowledge dominates selection at an early stage (and over the entire time-course of selection). Here then is an example of a study that used the same procedures as Theeuwes and colleagues and demonstrates (with a slightly different analysis) that top-down knowledge modulates early visual selection.

Interestingly and contrary to Theeuwes, saliency-based effects may not be purely bottom-up or early. One classical view is that stimuli with a high feature contrast enjoy a processing advantage because similar-looking items inhibit each other (via lateral inhibitory connections; e.g., Nakayama & Martini, 2011). Apart from this passive bottom-up advantage of salient stimuli (which can be overridden by top-down intentions; e.g., Eimer & Kiss, 2008), there is also some evidence that saliency may play a special role at later stages of visual processing.

In a recent EEG study, we found that a salient irrelevant distractor was not attended, yet could be recalled with higher accuracy than other non-salient distractors (Martin & Becker, in revision). This suggests that the visual system has evolved later mechanisms to *remember* salient items with greater accuracy (e.g., by allowing singletons preferential access to awareness/VSTM). Salient items are *potentially more informative* or *useful* for the visual system because they *can* be used for efficient orienting and thus may enjoy a top-down advantage. While further research is necessary to confirm these findings, it is very well possible that saliency plays a more important role for late processes mediating awareness than for early processes, and that these are in part top-down or strategic (e.g., Benoni, 2018; Kiss et al., 2012). Hence, directly reversing Theeuwes’ view on the timing and nature of top-down vs. bottom-up processes may yield a more accurate theory of attention and awareness than the currently prominent views.
